# Luminescent Properties of Zn and Mg Complexes on N-(2-Carboxyphenyl)salicylidenimine Basis

**DOI:** 10.3390/ma10080897

**Published:** 2017-08-03

**Authors:** Alexey Gusev, Elena Braga, Victor Shul’gin, Konstantin Lyssenko, Igor Eremenko, Lybov Samsonova, Konstantin Degtyarenko, Tatiana Kopylova, Wolfgang Linert

**Affiliations:** 1 General and Physical Chemistry Department, Crimean Federal University, Simferopol 295007, Russia; galex0330@gmail.com (A.G.); braga.yelena@ya.ru (E.B.); shulvic@gmail.com (V.S.); 2 A.N. Nesmeyanov Institute of Organoelement Compounds, Russian Academy of Sciences, Moscow 119991, Russia; kostya@ineos.ac.ru; 3 N.S. Kurnakov Institute of General and Inorganic Chemistry, Russian Academy of Sciences, Moscow 119991, Russia; ilerem@igic.ras.ru; 4 Laboratory of Organic Electronics, Tomsk State University, Tomsk 634050, Russia; slg@phys.tsu.ru (L.S.); norma1954@yandex.ru (K.D.); kopylova@phys.tsu.ru (T.K.); 5 Institute of Applied Synthetic Chemistry, Vienna University of Technology, A-1060 Vienna, Austria

**Keywords:** saliciylidenamide complex, Schiff base ligands, luminescence

## Abstract

New zinc and magnesium complexes of N-(2-carboxyphenyl)salicylidenimine) were synthesized and structurally characterized by elemental analysis, FT-IR, and X-ray single-crystal analysis. These complexes exhibit tuneable luminescence in the solid state from blue to green by varying by metal ion and composition. Moreover, the quantum yields range from 0.11 to 0.41, while lifetimes were determined to be in the nanosecond timescale. Thermal analysis shows that these complexes exhibit good thermal stability and can therefore well be used as electroluminescent materials.

## 1. Introduction

Research efforts on metal-coordinated organic materials with luminescent properties have been of great interest for decades because of their potential applications as components of electroluminescent diodes (OLED), lasers, and solar cells. The advantages of metal complexes with organic ligands as candidates for luminescent materials stem from the possibility of increasing the brightness and stability of emitters when a “purely” organic material is exchanged for a compound with optically inactive metal ions, such as zinc, aluminium, beryllium, etc. [1,2,3,4]. Of importance is the fact that luminescence of coordination compounds is a structure-sensitive property. Therefore, the observation of luminescence from these compounds can establish relationships between the composition and structure of organic metal complexes, their luminescence properties, and the properties of related materials [5].

Zinc complexes have been used in OLEDs for more than a decade [6,7,8], but the best electroluminescent performance of these materials as emitters is just comparable with that of tris-(8-hydroxyquinoline)aluminium (AlQ_3_). However, in many instances, the electron-transporting mobility of zinc complexes goes beyond then of AlQ_3_. Therefore, zinc complexes may be potential candidates to enhance the electron-transporting properties for OLEDs.

Schiff base ligands are frequently used in coordination chemistry due to their significant ability to form stable complexes with metal ions. Several highly efficient OLEDs have been already created based on zinc(II) complexes with Schiff bases. Interest in searching for new sources of blue luminescence is due to the fact that efficient EL materials, i.e., red and green luminophores, are already available among complexes and already used in OLEDs, whereas blue luminophores of comparable efficiency have hardly been found so far [9,10,11,12]. In addition, luminescent properties of zinc(II) complexes are determined only by the organic ligand because the d-shell of the central ion is completely filled. The luminescent properties of these compounds can be easily varied by changing the nature of substituents in the composition of the ligands [13].

**Scheme 1:**
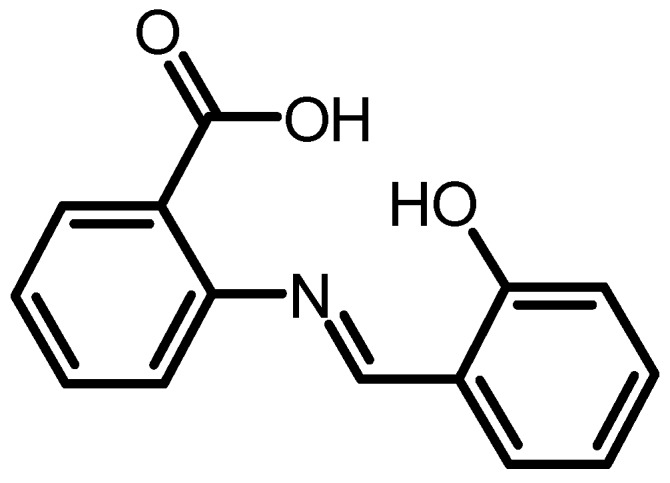
Structural formula of N-(2-carboxyphenyl)salicylidenimine.

Therefore, it would be helpful to design new promising fluorophores through a comparative study of optical properties of azomethine ligands with an understanding of substituent effects and the influence of structural features on emission behavior. This encouraged us to investigate and present the syntheses, characterization, and luminescent properties of homo- and heteroleptic complexes of Zn and Mg complexes of N-(2-carboxyphenyl)salicylidenimine (Scheme 1).

## 2. Results

### 2.1. General Characterization

**Scheme 2:**
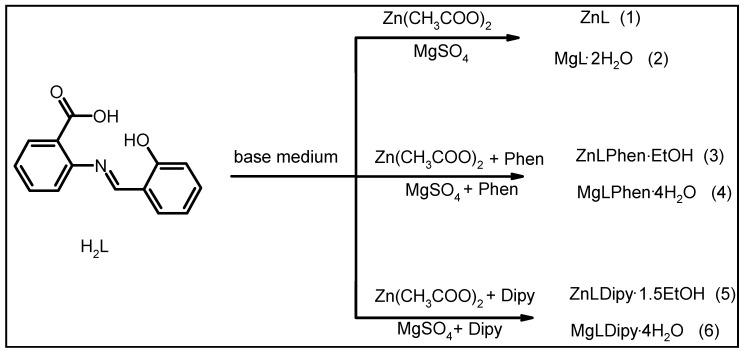
Synthesis of target complexes.

All the reported complexes were synthesized by the following sequential routes (Scheme 2).

The synthesized complexes were characterized by using different physiochemical techniques. The IR spectra of the zinc complexes exhibit the characteristic absorption bands of the ν(C–H), ν(C=N), ν(C–O) stretching vibrations, and the aromatic ring vibrations. The ν(C=N) band of the ligand at 1621 cm^−1^ is found to be shifted to lower energies (1593–1610 cm^−1^) in the spectra of the complexes, indicating coordination via the azomethine nitrogen. Valence stretching carboxylic vibration ν(C=O) of the protonated H_2_L at 1688 cm^−1^ is replaced by the asymmetric (1664–1619 cm^−1^) and the symmetric (1421–1361 cm^−1^) stretching of carboxylate-anion vibrations, whose positions are indicative of a monodentate coordinate mode of carboxylate group. The presence of ethanol or water molecules in the compounds 2–6 is indicated by a broad O–H stretching absorption band in the region 3450–3300 cm^–1^ [14].

Thermal stability is an important characteristic of the compounds to be used as the electroluminescent materials [9]. The thermal stability has been studied by thermogravimetric analysis (TGA). The zinc complex 1 is stable up to a temperature of 350 °C, a further increase of temperature lead to decomposition of 1 in the temperature range of 350–770 °C. Complexes 4 and 6 thermally decomposed in two steps. The first weight loss of 9% at around 90–200 °C for 3, and 11% at around 90–220 °C for 5 corresponding to the loss of the ethanol molecules. The desolvation of the title complexes leads to stable phases with thermal stability up to 280–320 °C, followed by a decay of the complexes via burning of the organic ligand. The process is completed at a temperature of 590–670 °C.

The TGA curves of Mg complexes 2, 4, and 6 are similar. Title complexes thermally decomposed in two steps. The weight loss at the temperature range of 90–190 °C associated with water molecules losing. Further heating over 270–340 °C leads to thermal oxidative degradation, transforming into a burning of the organic part of the complex.

### 2.2. Structures of Complex 5

**Figure 1:**
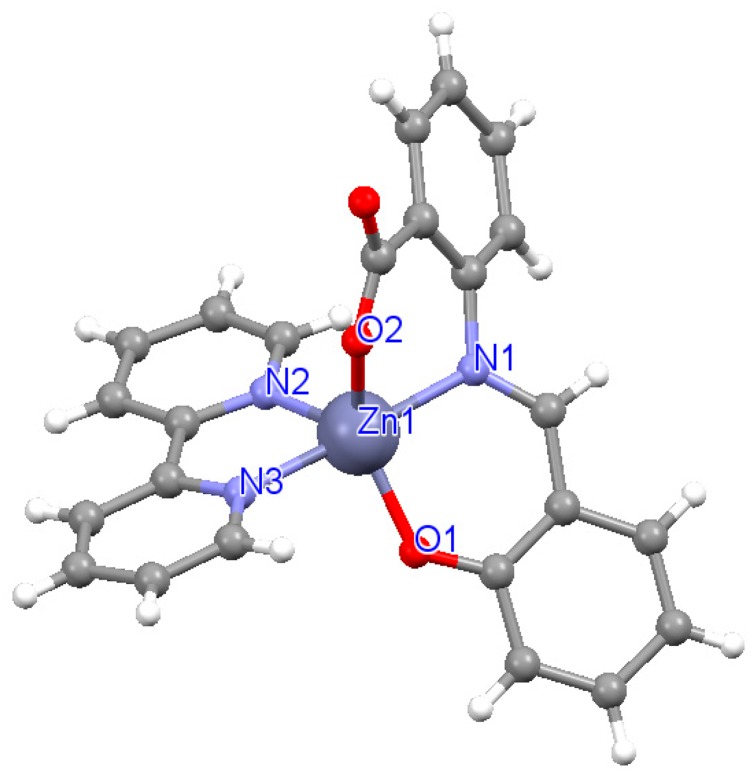
Structure of complex 1. Selected bond lengths (A) and bond angles (deg) are: Zn1-O1 1.9431(16), Zn1-O2 1.9937(16), Zn1-N1 2.0658(19), Zn1-N2 2.0925(19), Zn1-N3 2.1228(18), O1-Zn1-O2 129.76(7), O1-Zn1-N1 91.29(7), O2-Zn1-N1 86.41(7), O1-Zn1-N2 121.05(7), O2-Zn1-N2 108.32(7), N1-Zn1-N2 102.62(7), O1-Zn1-N3 90.55(7), O2-Zn1-N3 91.01(7), N1-Zn1-N3 177.41(7), N2-Zn1-N3 77.96(7).

Crystalline and molecular structure of 1·MeOH were described yearly by W.-Z. Ju et al. [15]. According to studies, complex has a polymeric structure. Light-yellow crystals of the complex ZnLDipy·EtOH suitable for X-ray diffraction studies were grown by slow evaporation of a solution of the bulk complex in CH_3_CN. The crystallographic and refinement data are listed in Table 1. A perspective drawing of the structure is shown in Figure 1. X-ray single-crystal structural analysis reveals that complex 5 crystallizes in the triclinic space group $P\bar{1}$ and the asymmetric unit includes independent complex and MeCN solvent molecules. Zinc structure consists of a tridentate ONO- donor Schiff base ligand in deprotonated form and bidentate 2,2-dipyridine. The bond distances of the donor atoms of the Schiff base ligand to the central metal atom for these complexes are in the range 1.943 to 2.066 Å and the bond distances of the dipyridine ligand to the metal atom are 2.092 and 2.123 Å. The zinc ion is five-coordinate and has distorted trigonal bipyramidal geometry (τ = 0.8). Schiff base chelating double rings are noticeably bent, forming an angle 40.82° between best-fit planes.

### 2.3. Photophysical Studies

**Figure 2:**
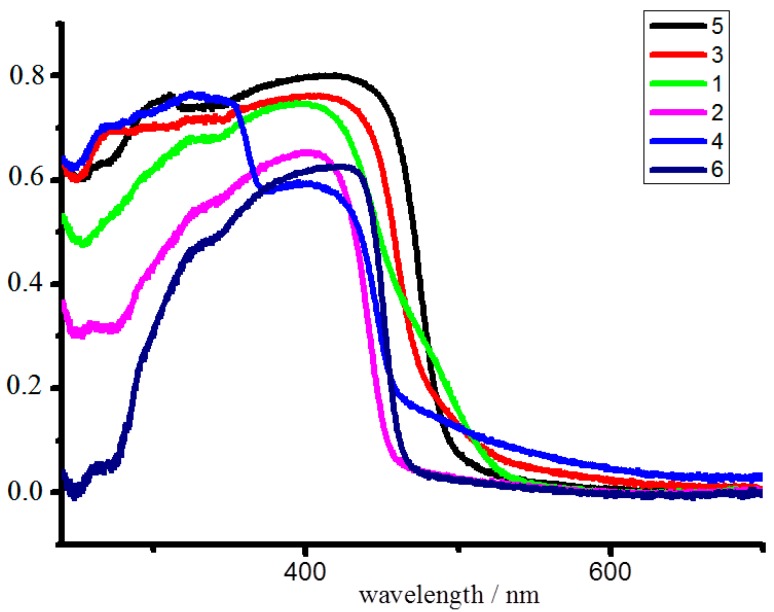
Reflectance spectra of 1–6.

From previous studies, it is known that the luminescent properties of thin films and solid samples are similar [9]. Since the title complexes are considered as candidates for thin-film electroluminescent materials, they have been studied concerning their photo-physical characteristics using solid samples. The reflectance spectra of 1–6 are quite similar (Figure 2) and exhibit two main bands at 311–331 nm assigned to π–π* transitions of the aromatic parts and 399–424 nm originating for the metal perturbed intraligand π–π* transition of the C=N azomethine and phenolate units.

**Figure 3:**
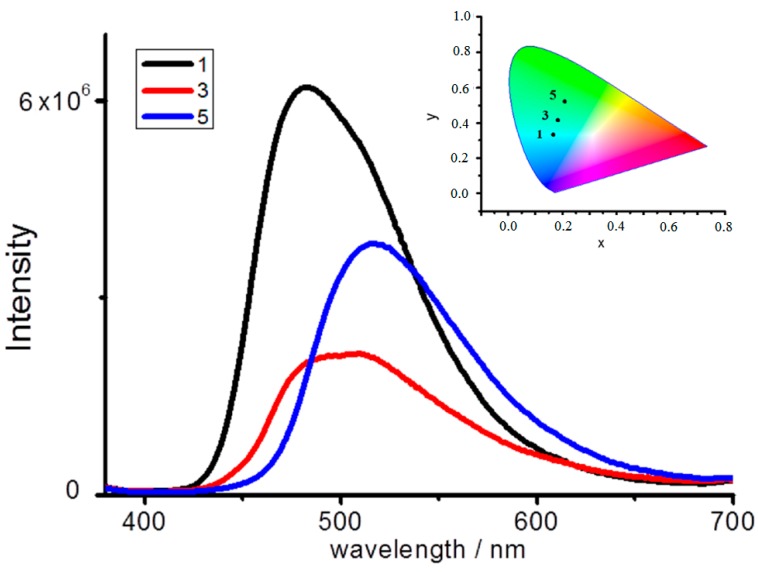
Emission spectra of zinc complexes in solid state and the corresponding color coordinate diagram of emission.

**Figure 4:**
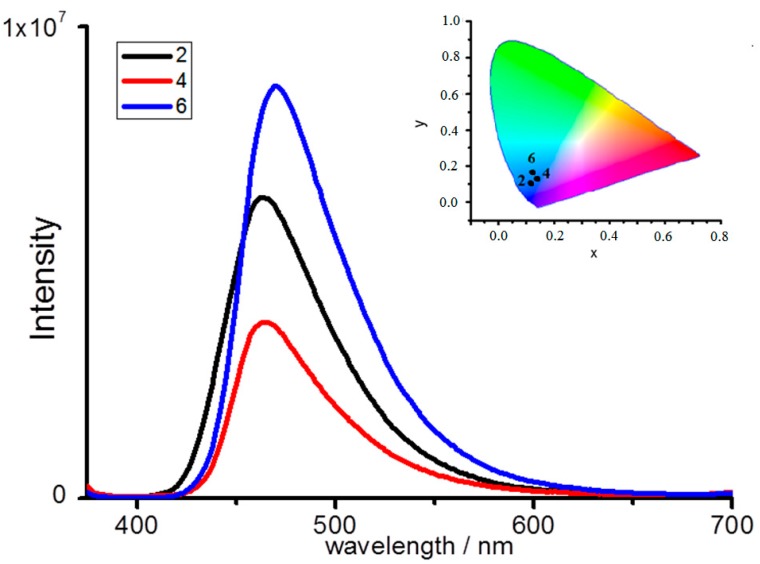
Emission spectra of magnesium complexes in solid state and the corresponding color coordinate diagram of emission.

Luminescent zinc complexes possessing closed electronic shells are superior potential candidates as valuable luminescent materials; thus, we probed the luminescence properties of 1–6 in the solid state. For the zinc complexes 1, 3, and 5 the emission bands are observed at 482, 508, and 515 nm (Figure 3), respectively. These luminescence emissions are all attributable to the ligand-centered π*–π transitions. For Mg complexes 2, 4, and 6 emission bands are observed at 463, 464, and 469 nm, respectively (Figure 4). Coordination of 2,2-dipyridine or 1,10-phenantroline occur red-shifting of emission band. Addition of heterocyclic ligands probably leads to the appearance of additional molecular levels, which leads to a decrease in the energy gap between HOMO and LUMO and a bathochromic shift of luminescent maxima. Luminescent bands of Zn complexes bands exhibit greenish-blue luminescence while Mg complexes exhibit deep blue luminescence emissions, and the Commission Internationale d’Eclairage (CIE) coordinates are summarized in Table 2.

The quantum yields of all ligands and complexes have been determined for solid samples at 298 K (Table 2). It was found that the quantum yields of the homoleptic complexes 1 (QY = 0.312) and 2 (QY = 0.399) are higher than those of the free ligand (0.002). This is because the metal centers in complexes play a significant role in enhancing the ligand-centered π*–π fluorescent emission. The chelation of the ligand to the metal center could increase the rigidity of the ligand, and reduce the loss of energy by thermal vibrational decay. Coordination of 2,2-dipyridine or 1,10-phenantroline to zinc ion leads to a decrease of luminescent efficiency, this is probably due to the appearance of additional energy loss pathways. For Mg analogs maximal quantum yield was found for complex 6. Generally quantum efficiency for Mg complexes are higher that Zn analogs. Although an enhancement of the quantum yield of luminescence was observed for Schiff base ligand, no significant variation of the luminescence lifetime was observed. In particular, the lifetimes were determined to be in the nanosecond timescale, which is consistent with values previously determined for fluorescent zinc complexes [13].

In this way, high luminescence efficiency in the green and blue light region, as well as the high thermally stability, indicates that the two complexes may be excellent candidates for highly thermally stable fluorescent materials.

**Figure 5:**
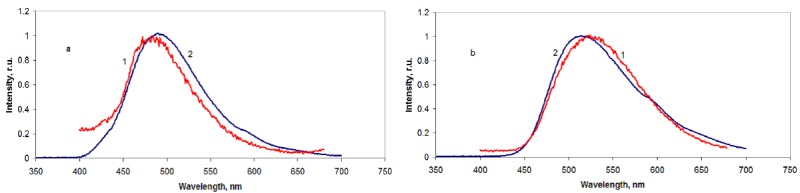
PL (1) and EL (2) spectra of complexes 5 (**a**) and 6 (**b**).

The title complexes 5 and 6 were used to develop a prototype for electroluminescent devices. The electroluminescent (EL) properties were studied for simple sandwich structures ITO/PEDOT:PSS/NPD/complex/LiF/Al by using complexes 5 and 6 as an emissive layer in the configuration. Both complexes emit in the applied voltage. The electroluminescence spectrum of each of these compounds practically coincides with the corresponding photoluminescence spectrum for thin films obtained by thermo-vacuum deposition (Figure 5). It indicates that the EL and PL have the same origin. The maxima of the EL complexes of 5 and 6 at λ = 515 and 492 nm, respectively.

**Figure 6:**
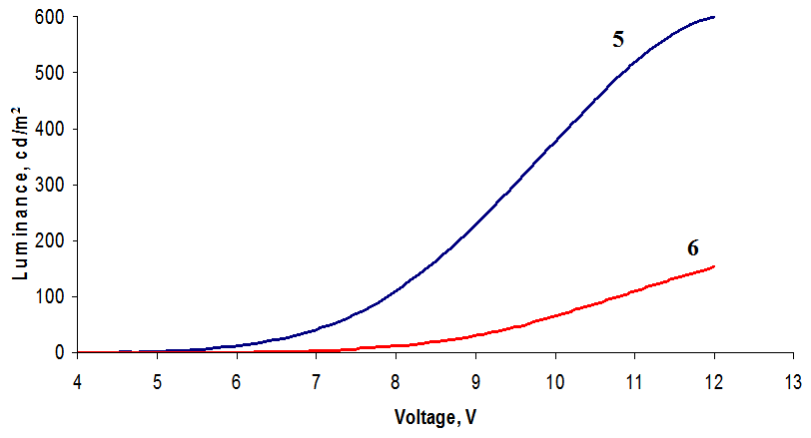
Luminance-voltage characteristics of the OLED devices.

The EL brightness as a function of the applied voltage is presented in Figure 6. Emission for 5 was observed at as low as 5.5 V and for 6 at as low as 7 V. The brightness of the devices increases with the increasing of bias voltage. Notably, those EL spectra were independent of the bias voltage. It can be seen that the brightness of OLED emission based on 5 is higher than on the basis of 6, despite the fact that the quantum yield of photoluminescence is in the opposite ratio (Table 1). The maximum brightness of the ITO/PEDOT:PSS/NPD/5/LiF/Al device is about 600 cd/m^2^ at a driving voltage of 12 V while maximum brightness for ITO/PEDOT:PSS/NPD/6/LiF/Al reach only 120 cd/m^2^. The point is that the effectiveness of OLED is influenced by many factors, among which is the charge transport properties of emitting layer, as well as the energy balance of the auxiliary layers of the OLED structure. Obviously, when the second condition is fulfilled, the brightness properties of these complexes will be much higher. Research to find the optimal structure for maximum brightness is currently in progress.

## 3. Materials and Methods 

All starting reagents and chemicals were purchased from Aldrich or Merck and used without further purification. Solvents used for spectroscopic studies were purified and dried by standard procedures before use. Reflectance UV-Vis spectra were recorded on a Cintra 4040 spectrophotometer. Luminescence spectra both of solutions and of solid samples were recorded on a FluoroMax-4 spectrofluorimeter. Solid sample quantum yields were determined under ligand excitation using an integrating sphere absolute method. Lifetime measurements were performed on a Horiba Fluorocube lifetime instrument by a time-correlated single-photon counting method using a 365 nm LED excitation source. Elemental analyses of C, H, and N were performed with a Perkin-Elmer 240 C analyzer. IR spectra were measured with a FSM 2202 spectrometer with KBr pellets in the range 4000–400 cm^–1^. Thermogravimetric experiments were performed on a Paulik-Paulik-Erdey Q-derivatograph under static air atmosphere (see Table 3).

Single crystal structure determination by X-ray diffraction was performed on a Bruker Apex-II CCD diffractometer (МоКα radiation, graphite monochromator, λ = 0.71073 Å) at 298 K. The refined cell constants and additional relevant crystal data are given in Table 1. The structures were solved by the direct method and refined by full-matrix least-squares in the anisotropic approximation (SHELX-97) [16,17]. Crystallographic data for compound 5 have been deposited with the Cambridge Crystal Database (CCDC number 1556748).

The homoleptic Zn complexes were synthesized according literature method [15]. The homoleptic Mg complex was synthesized according following procedure. A mixture of salicylaldehyde and 2-aminobensoic acid in a 1:1 molar ratio was heated in ethanol (30 mL) for 1 h. After that, solution MgSO_4_·7H_2_O (1 mmol) in 10 mL of water was gradually added to reaction solution. After the mixture was stirred for 0.5 h, 2 mL of 1 M NaOH solution was added and stirred again for 2 h at room temperature. Light-yellow precipitate was produced during reaction. The crude product was collected by filtration and washed with ethanol and finally dried on air.

The heteroleptic Zn and Mg complexes were obtained in similar manner by addition of equimolar amounts of 2,2-dipyridine or 1,10-phenantroline to homoleptic complexes so method of preparation of ZnLDipy is presented only. To a EtOH suspension of corresponding homoleptic complex (1 mmol), equimolar amounts of 2,2-dipyridine were added. Reaction mixture was stirred for 30 min at 50–60 °C. After cooling to ambient temperature, crystalline solid was formed. The precipitate was separated by filtration and dried on air.

Creation of OLED structures: glass substrates coated with a transparent layer of a mixture of indium and tin oxides (ITO) with a resistance of 12 Ω/sq were used. Preliminary preparation of substrates was carried out according to the established procedure: thorough purification in organic solvents with subsequent etching in oxygen plasma. The application of the layers to the prepared substrate was carried out in a glovebox under dry nitrogen atmosphere. Thermovacuum deposition (TVD) was performed on the “AUTO 306” equipment by “BOC EDWARDS” (Crawley, UK) using shadow masks at a residual pressure of ~10^−5^ mbar and with deposition rates of organic layers 0.2 nm/s, metals 2 nm/s. The emission areas were 4 × 4 mm^2^. The layers of organic substances and cathode metals were formed without depressurizing chamber. The evaporation speed and thickness of the deposited layers were controlled by a quartz detector SQM 160 (INFICON GmbH, New York, NY, USA).

The voltage-current, voltage-brightness, and spectral characteristics of the obtained OLED structures were studied on a measuring complex consisting of a voltage analyzer source (Keithley 237, KEITHLEY, Cleveland, OH, USA) and a fiber spectrometer (AvaSpec-ULS-2048 × 64, Avantes BV, Apeldoorn, The Netherlands).

For the study of electroluminescent properties, samples of the following composition were prepared: ITO/PEDOT:PSS/NPD/complex/LiF/Al. PEDOT:PSS (AI 4083 Heraeus Clevios)-2.8 wt % aqueous solution of poly(3,4-ethyldioxythiophene): poly(styrenesulfonate) (Sigma-Aldrich, St. Louis, MO, USA) was applied to ITO by spin coating at 4000 rpm for 30 s, and dried at a temperature of 120 °C. This substance is used not only to smooth the surface of the anode, but is also an emitter of holes. The thickness of the PEDOT:PSS films was 30 nm.

NPD-N4,N4′-di(naphthalen-1-yl)-N4,N4′-diphenylbiphenyl-4,4′-diamine was deposited by the TVD method. The thickness of the layer was 20 nm. This substance in sandwich structures acts as of hole transport material.

We used complexes 5 and 6 as an emitting layer. The thickness of the layer was 40 nm.

LiF with a thickness of 1 nm is used both to lower the energy barrier between the LUMO level of the complexes and the work function of the electron from aluminium, and to prevent the introduction of aluminium atoms into the radiating layer during the formation of the cathode. Al served as a cathode.

## 4. Conclusions

In summary, six novel homo- and heteroleptic complexes of Zn(II) and Mg(II) complexes of N-(2-carboxyphenyl)salicylidenimine have been successfully assembled and well characterized. The results presented herein indicate that title complexes demonstrate high luminescence in solid state and can be used as emitting materials in electroluminescent diodes.

## Figures and Tables

**Table 1:** Crystallographic and structural determination data for complex 5.

Parameter	Complex 5	
Formula	C_26_H_20_N_4_O_3_Zn	
Crystal system	triclinic	
Space group	$P\bar{1}$	
Unit cell parameters	а, Å b, Å c, Å V, Å^3^ α, ° β, ° γ, ° Z	9.1160(8) 9.9081(9) 12.8594(12) 1098.11(17) 77.375(2) 80.345(2) 77.619(2) 2
P_calc._ g⋅cm^−3^	1.518	
μ_Mo_. mm^−1^	1.156	
F(000)	592	
θ_max_. deg	2.143–28.997	
Index ranges	−12 ≤ h ≤ 12 −13 ≤ k ≤ 13 −17 ≤ l ≤ 17	
Reflections measured/reflections independent	5860/4570	
GOOF	1.008	
R (all data)	R_1_ = 0.0574 wR_2_ = 0.1077	
R(I > 2σ(I))	R_1_ = 0.0406 wR_2_ = 0.0986	
Residual electron density (max/min). е/Å^3^	0.885/−0.282	

**Table 2:** UV–Vis, photoluminescence spectra data for title complexes in solid state.

Complex	Absorbance λ_max_ (nm)	Emission λ_max_ (nm)	Quantum Yield (%)	Lifetime (ns)	CIE Coordinates
1	323, 399	482	31.2	3.6	0.172; 0.344
2	326, 401	463	39.9	4.2	0.142; 0.155
3	324, 406	508	11.0	2.6	0.235; 0.461
4	328, 402	464	19.0	3.6	0.143; 0.179
5	311, 416	515	16.9	3.1	0.281; 0.584
6	331, 424	469	41.4	3.8	0.141; 0.208

**Table 3:** Basic properties of the prepared complexes.

Complex		Property	Yield (%)	FTIR (KBr, cm^−1^)	Element Anal. Calc. (%)	Found (%)
1	ZnL (C_14_H_9_NO_3_Zn)	light-yellow solid	85	1627, 1601, 1539, 1451, 1411, 1304, 1179, 743, 715, 542	C, 55.20; H, 3.98; N, 4.59	C, 55.06; H, 3.96; N, 4.80
2	MgL·2H_2_O (C_14_H_13_MgNO_5_)	light-yellow solid	51	3405, 2794, 1660, 1607, 1532, 1470, 1418, 1333, 1179, 1155, 1127, 900, 753, 730, 660, 509	C, 56.13; H, 4.37; N, 4.68	C, 55.52; H, 4.86; N, 4.42
3	ZnLPhen·EtOH (C_28_H_23_N_3_O_4_Zn)	yellow solid	74	3415, 1622, 1593, 1548, 1448, 1368, 1309, 1179, 1151, 847, 764, 727	C, 63.34; H, 4.37; N, 7.92	C, 63.17; H, 4.51; N, 7.94
4	MgLPhen·4H_2_O (C_26_H_25_MgN_3_O_7_)	yellow solid	41	3431, 3329, 1664, 1610, 1572, 1532, 1456, 1385, 1302, 1177, 1158, 1124, 840, 762, 727	C, 60.54; H, 4.88; N, 8.15	C, 60.11; H, 5.38; N, 8.32
5	ZnLDipy·1.5EtOH (C_27_H_26_N_3_O_4.5_Zn)	yellow solid	68	3438, 1619, 1601, 1560, 1530, 1447, 1361, 1316, 1174, 1147, 1025, 866, 764, 719	C, 61.24; H, 4.91; N, 7.93	C, 61.12; H, 4.70; N, 8.04
6	MgLDipy·4H_2_O (C_24_H_25_MgN_3_O_7_)	light-yellow solid	42	3464, 3187, 2797, 1664, 1605, 1534, 1475, 1421, 1335, 1180, 1158, 1124, 902, 757, 734, 663	C, 58.61; H, 5.12; N, 8.54	C, 58.21; H, 4.98; N, 8.22

## References

[1] Wang S. (2001). Luminescence and electroluminescence of Al(III), B(III), Be(II) and Zn(II) complexes with nitrogen donors. Coord. Chem. Rev..

[2] Evans R.C., Douglas P., Winscom C.J. (2006). Coordination complexes exhibiting room-temperature phosphorescence: Evaluation of their suitability as triplet emitters in organic light emitting diodes. Coord. Chem. Rev..

[3] Yam V.W.-W., Au V.K.M., Leung S.Y.-L. (2015). Light-Emitting Self-Assembled Materials Based on d^8^ and d^10^ Transition Metal Complexes. Chem. Rev..

[4] Rezaeivala M., Keypour H. (2014). Schiff base and non-Schiff base macrocyclic ligands and complexes incorporating the pyridine moiety–The first 50 years. Coord. Chem. Rev..

[5] Kuz’mina N.P., Eliseeva S.V. (2006). Photo and Electroluminescence of Lanthanide(III) Complexes. Russ. J. Inorg. Chem..

[6] Xie J., Qiao J., Wang L., Xie J., Qiu Y. (2005). An azomethin-zinc complex for organic electroluminescence: Crystal structure, thermal stability and optoelectronic properties. Inorg. Chim. Acta.

[7] Jang Y.-K., Kima D.-E., Kim W.-S., Kwon O.-K., Lee B.-J., Kwon Y-S. (2006). Electroluminescent properties of organic light emitting diodes using Zn(HPB)_2_ and Zn(HPB)_q_. Colloids Surf. A Physicochem. Eng. Asp..

[8] Son H.-J., Han W.S., Chun J.-Y., Kang B.-K., Kwon S.-N., Ko J., Han S.J., Lee C., Kim S.J., Kang S.O. (2008). Generation of Blue Light-Emitting Zinc Complexes by Band-Gap Control of the Oxazolylphenolate Ligand System: Syntheses, Characterizations, and Organic Light Emitting Device Applications of 4-Coordinated Bis(2-oxazolylphenolate) Zinc(II) Complexes. Inorg. Chem..

[9] Kotova O.V., Eliseeva S.V., Averjushkin A.S., Lepnev L.S., Vaschenko A.A., Rogachev A.Y., Vitukhnovskii A.G., Kuzmina N.P. (2008). Zinc(II) complexes with Schiff bases derived from ethylenediamine and salicylaldehyde: The synthesis and photoluminescent properties. Russ. Chem. Bull. Int. Ed..

[10] Deda M.L., Ghedini M., Aiello I., Grisolia A. (2004). A New Blue Photoluminescent Salen-like Zinc Complex with Excellent Emission Quantum Yield. Chem. Lett..

[11] Gusev A.N., Shul’gin V.F., Konnic O.V., Meshkova S.B., Aleksandrov G.G., Kiskin M.A., Eremenko I.L., Linert W. (2011). New Zn complexes based on 1,2,4-triazoles: Synthesis, structure and luminescence. Inorg. Chim. Acta.

[12] Chen L., Qiao J., Xie J., Duan L., Zhang D., Wang L., Qiu Y. (2009). Substituted azomethine–zinc complexes: Thermal stability, photophysical, electrochemical and electron transport properties. Inorg. Chim. Acta.

[13] Dumur F., Contal E., Wantz G., Gigmes D. (2014). Photoluminescence of Zinc Complexes: Easily Tunable Optical Properties by Variation of the Bridge between the Imido Groups of Schiff Base Ligands. Eur. J. Inorg. Chem..

[14] Ligtenbarg A.G.J., Hage R., Meetsma A., Feringa B.L. (1999). Hydrogen bonding properties and intermediate structure of N-(2-carboxyphenyl)salicylidenimine. J. Chem. Soc. Perkin Trans..

[15] Ju W.-Z., Shi L., Chen K., Xue J.-Y. (2005). Catena-Poly[[zinc(II)-µ-[2-(2-carboxylatophenyliminomethyl)phenolato-1k^3^O,N,O’:1’kO’’]]methanol solvate]. Acta Cryst..

[16] (1997). SHELXL97; Program for the Refinement of Crystal Structures.

[17] Sheldrick G.M. (2008). A short history of SHELX. Acta Crystallogr..

